# Current Practice of Occupational Therapy for Common Disorders Seen in Rehabilitation Clinics in Saudi Arabia

**DOI:** 10.1155/oti/5192064

**Published:** 2025-07-15

**Authors:** Alaa M. Arafah, Samar Altherwy, Seham Alzahrani, Ghadeer Alghamdi, Roaa Alghamdi, Mannar Haddad

**Affiliations:** ^1^Department of Occupational Therapy, Faculty of Medical Rehabilitation Sciences, King Abdulaziz University, Jeddah, Saudi Arabia; ^2^Department of Speech Language Pathology and Audiology, Faculty of Medical Rehabilitation Sciences, King Abdulaziz University, Jeddah, Saudi Arabia

**Keywords:** common conditions, common interventions, occupational therapy (OT), rehabilitation, Saudi Arabia

## Abstract

**Background:** The role of occupational therapy is to provide management for various conditions including neurological, musculoskeletal, and psychological disorders, with the aim of maximizing function and independency in daily occupations. According to the World Federation of Occupational Therapists (WFOT), occupational therapy is a client-centered health profession concerned with promoting health and well-being through occupation. In Saudi Arabia, there are escalating rates of chronic conditions, which create an increasing demand for occupational therapy services. Yet, occupational therapy practice frameworks, as well as areas of assessment and intervention, are not well explored within the context of Saudi Arabia.

**Purpose:** The aim of this research is to identify the most common disorders that occupational therapists work with within Saudi Arabia and the approaches used for managing these disorders.

**Methods:** This was a cross-sectional study. An electronic survey was distributed to 230 occupational therapists working in Saudi Arabia. The survey consisted of two sections; the first was on educational backgrounds, and the second section was about the common conditions encountered in a clinical setting and the treatment approaches applied. The interventions nominated by participants were mapped to the person–environment–occupation model and to the occupational therapy practice framework.

**Results:** The overall response rate was 57%. Analysis of data of 131 participants revealed that neurological diseases (e.g., stroke, multiple sclerosis, and Parkinson's disease) were the most common conditions managed by occupational therapists (64.3%), while respiratory diseases had the lowest percentage (4.7%). With regard to intervention approaches, “therapeutic exercise” was the most commonly applied approach as was reported by 77.1% of the therapists, while “functional electrical stimulation” was the lowest chosen approach as it was applied by 19.8% of the therapists.

**Conclusion:** Occupational therapists in Saudi Arabia manage a variety of conditions and apply a wide range of rehabilitation approaches, yet gaps still exist in providing a holistic approach. The study emphasizes the importance of redirecting the focus of occupational therapists to core concepts of maximizing functioning and occupational performance and using occupation as a mean and as an outcome of rehabilitation.

## 1. Introduction

Occupational therapy (OT) is a medical rehabilitation discipline that enables individuals to perform daily activities and improve their quality of life [1]. OT helps individuals of all ages overcome diseases and disabilities, including neurological conditions (e.g., stroke, multiple sclerosis, and traumatic brain injury [TBI]), orthopedic conditions (e.g., fractures, rheumatoid arthritis, and osteoarthritis), mental health disorders (e.g., depression, anxiety disorders, and schizophrenia), developmental disorders (e.g., autism, Down syndrome, and cerebral palsy), and geriatric disorders (e.g., Parkinson's disease, Alzheimer, and dementia) [2, 3].

The overall goal of OT is to maximize functioning and independency in occupational performance [4]. According to the World Federation of Occupational Therapists (WFOT), OT is a client-centered health profession concerned with promoting health and well-being through occupation [5]. Across the different physical and mental disability cases, OT is aimed at improving the functioning of individuals at home [6] and within their communities [7, 8]. OT plays a vital role in improving occupational performance, where occupations cover three main domains of self-care (dressing, grooming, eating, etc.), productivity (studying, working, volunteering, etc.), and leisure (swimming, gardening, reading, etc.) [4].

Day-to-day activities, deemed meaningful and worthwhile by individuals, fall under the purview of OT. According to the American Occupational Therapy Association (AOTA), *domains* defining the focus of OT should incorporate all services that allow clients to engage in daily activities in selected contexts, roles, and circumstances at the person, group, or population levels. In addition to these domains, the AOTA outlines the service delivery *process*, including valid and reliable assessments, evidence-based interventions, and occupation-based individualized outcomes [1]. Within this scope, OT services may include but are not limited to hand, cognitive, mental, sensory, work, driving, and burn management.

OT interventions vary in their approaches. As outlined by the AOTA, approaches to OT intervention include remediation (improving impaired functions), compensation (adapting tasks or environment), rehabilitation (restoring lost skills), and health promotion/prevention (preventing the onset of occupational performance issues by promoting healthy habits and routines) [1]. These approaches are used based on the nature of the condition and the individual's goals. Across physical and mental health conditions, OT supports individuals at home, in educational and vocational settings, and in community participation.

Similar to other medical rehabilitation disciplines, OT is based on scientific evidence. These services are effective for diverse neurological, musculoskeletal, and psychosocial conditions covering all age groups [9]. Based on an extensive literature review, several researchers have reported distinct treatment strategies for various conditions. More specifically, while some researchers agreed on employing a specific method for a certain condition, others suggested different methods for similar conditions [3, 10–15].

Among the treatment strategies for neurological conditions such as stroke, the use of assistive technologies—including ramps and electrical hoists for transportation—has been found to be effective [3, 10, 11]. Another widely used intervention is constraint-induced movement therapy (CIMT), which involves restricting the use of the unaffected (i.e., unimpaired) limb to encourage use of the more affected (i.e., weaker) limb during functional activities [10].

For orthopedic conditions such as trigger finger—identified as the second most commonly addressed condition in the literature—the application of splints is a frequently recommended intervention [11, 12]. In addition, other treatment modalities such as orthoses, laser therapy, fluidotherapy, and specific therapeutic exercises have also been suggested [13, 14].

In the management of pediatric conditions such as Down syndrome and autism spectrum disorder, sensory integration therapy is commonly emphasized to enhance participation in daily occupations [15, 16]. One study compared the effectiveness of sensory integration alone to a combined approach involving sensory integration, vestibular stimulation, and neurodevelopmental therapy. Results indicated that the combined approach yielded better outcomes than sensory integration alone [16]. In contrast, another study reported that compensatory techniques and contextual modifications were the most frequently used interventions for individuals with Down syndrome [17].

Cerebral palsy is another condition commonly addressed in OT, as reported by Rassafiani et al. [18]. Recommended interventions often include active vestibular activities, such as the use of a bolster swing, therapy ball, ramps, or trampoline, to improve balance and coordination [18, 19]. Additionally, hippotherapy has demonstrated effectiveness in enhancing postural control and balance in children with cerebral palsy [20].

In the domain of mental health, OT plays a vital role in promoting functional independence, emotional regulation, and engagement in meaningful daily activities. OT interventions for individuals with mental health conditions, such as depression, and anxiety disorders, often focus on enhancing coping strategies, building social skills, and improving participation in self-care, work, and leisure activities [21].

One common approach involves the use of cognitive behavioral strategies to address distorted thought patterns and support behavior modification, particularly for individuals experiencing anxiety or mood disorders [22]. Group-based interventions, including life skill training and psychoeducation, are also frequently employed to improve interpersonal functioning and promote community integration [23].

It should be noted that OT practice is influenced by socioeconomic conditions, values, and cultures [24]. Previous research indicated that creating a culture with diverse meanings and occupation-focused practices requires consideration of various worldviews [25, 26]. Rehabilitation and disability services in Saudi Arabia have undergone significant transformation in recent years, emphasizing the inclusivity of individuals with disability. Within this evolving landscape, OT has emerged as a vital component of the rehabilitation sector. OTs are formally certified by the Saudi Commission for Health Specialties (SCFHS) and contribute to patient care through assessment, treatment planning, and therapeutic interventions. OT services in Saudi Arabia are primarily delivered within secondary and tertiary healthcare settings, focusing on patients with physical, neurological, and developmental conditions. According to recent Ministry of Health statistics, over 91,000 patients received OT services from ministry sectors in 2022 alone, with an additional 59,000 served by other government bodies, such as military and educational hospitals [27]. A recent study on the scope of OT practice in Saudi Arabia revealed that services extend beyond hospital-based rehabilitation to include autism centers, facilities for children with disabilities, and home healthcare programs. However, OT services remain largely absent in primary care, school settings, and community-based programs, indicating a critical area for future development. Moreover, OT services in Saudi Arabia typically require a referral from a physician, which reflects the current medical model of care [28]. While this model ensures collaboration, it also highlights the limited autonomy of OTs in clinical decision-making and underscores the need for stronger interdisciplinary coordination.

Although OT services can be accessed at major rehabilitation hospitals, their availability in academic settings in Saudi Arabia has only been established within the past 13 years [29]. OT is being taught at only five universities in Saudi Arabia. The programs are united in several aspects, where students are enrolled in a 4-year course-based education followed by 1 year of internship. Most programs offer OT at an undergraduate level, where a bachelor's degree is awarded upon the completion of the program. The majority of the programs start with courses of basic medical sciences of anatomy, physiology, and biomechanics, followed by more OT core courses for assessing and treating different pediatrics, geriatrics, neurologic, musculoskeletal, and psychiatric conditions [29].

In addition to the novelty of the OT profession in Saudi Arabia, there is a lack of awareness among health professionals and community members in Saudi Arabia regarding the most frequent conditions and effective treatment procedures provided by occupational therapists [30–34], which can hinder the identification of patients needing OT. Moreover, the limited knowledge of the role of OT among health professionals may confine treatment approaches that could maximize patients' quality of life within a multidisciplinary rehabilitation approach. An in-depth understanding of common disorders encountered at OT clinics and corresponding treatment approaches will aid in improving the OT services provided. Moreover, closing this gap in knowledge will contribute to the awareness of the OT field in the Kingdom of Saudi Arabia and help enhance and develop this profession at both academic and clinical levels.

Given the abovementioned context, it is essential to identify the most prevalent conditions managed by occupational therapists in Saudi Arabia, as well as the therapeutic strategies commonly employed in their treatment. Such an investigation holds the potential to enhance both public and professional awareness, promote the expansion of OT services, and support the advancement of the profession at academic and clinical levels. Although international literature has extensively documented common OT interventions across a range of conditions [3–20], there remains a paucity of local data specific to the Saudi context.

Accordingly, the present study is aimed at determining the most frequently utilized interventional techniques and the conditions for which OT is most commonly sought in Saudi Arabia. The findings are anticipated to raise awareness among community members and healthcare professionals regarding the scope of OT, including the common disorders addressed and the treatment approaches implemented. Furthermore, the study seeks to contribute to the existing body of knowledge by providing context-specific insights that may inform the practices of occupational therapists across the country.

## 2. Materials and Methods

### 2.1. Study Design

This cross-sectional study involved an electronic survey, which was conducted from February 2022 to May 2022 and distributed to occupational therapists working in Saudi Arabia.

### 2.2. Participants

The study included occupational therapists that met the following characteristics: attained at least a bachelor's degree in the field of OT, has at least 1 year of experience in the clinical field (school settings, university clinics, specialized centers, acute care settings, outpatient clinic centers, inpatient settings, and rehabilitation centers), living in Saudi Arabia, certified by the SCFHS, and speak Arabic or English. The study excluded occupational therapists who had graduated recently with less than a year of clinical experience.

### 2.3. Materials and Procedure

This study was approved by the Faculty of Medical Rehabilitation Sciences Ethical Committee at King Abdulaziz University (FMRS-EC2022-008). The study consisted of a self-administered survey targeting occupational therapists in Saudi Arabia using convenience sampling techniques. The main authors contacted the Saudi Occupational Therapy Association, as well as certain pioneering professionals in the field of OT, to help in the recruitment of occupational therapists in Saudi Arabia by ascertaining their email addresses and contact information. The study pamphlet was then directly sent to identified occupational therapists (*n* = 230). In addition, convenience sampling techniques were used where social media platforms relating to OT and rehabilitation were used to recruit occupational therapists across different geographical regions of Saudi Arabia. Once occupational therapists agreed to participate in the study, they were guided to the link to the survey.

The study survey consisted of two sections. The first section included questions regarding educational background and personal information, such as gender, years of experience, qualifications, and geographical information. The second section included questions on the main study outcomes and was further divided into two categories: the first concerned common conditions that the participant had encountered in his/her clinical setting, while the second encompassed questions regarding treatment methods employed to treat the relevant condition. Questions included “other” as an optional answer to allow participants to provide an appropriate answer if a suitable answer was not listed among the choices. The questionnaire was reviewed by two OT experts and pilot-tested by five individuals to overcome barriers to understanding or completing the questionnaire. Google Forms was the primary platform used for data collection.

### 2.4. Statistical Analysis

Descriptive statistics were used for data analysis, with categorical data presented as percentages and frequencies and continuous data as means and standard deviations. Qualitative data for the open-ended questions were presented as nominal data according to the common themes and patterns gathered from the participants' responses. Interventions nominated by the participants were mapped to the person–environment–occupation (PEO) model [35]. The PEO model was created as a framework for services that utilize client-centered approaches. The model comprises three parts: the person, environment, and occupation. A person possesses a distinctive set of identities, abilities, and skills. Physical, social, cultural, and socioeconomic elements fall under the broad environmental category. The functional activities a person performs are referred to as their occupation. The level of a person's capabilities relative to their functioning level determines the relevance of PEO contact. Thus, the results of the current study were mapped to the PEO model to indicate the extent to which OT services in Saudi Arabia align with this best practice model.

## 3. Results

Overall, 131 occupational therapists participated in the survey with a response rate of 57%, comprising 80 (61.1%) females and 51 (38.9%) males.  Table 1 summarizes the sociodemographic and educational characteristics of the participants. Most participants (88.5%) held a bachelor's degree, and 9.2% also held a master's degree.  Table 1 presents the remaining analysis of participants' qualifications.

As presented in Table 1, the majority of participants (*n* = 86, 64.9%) had 1–3 years of experience, whereas only 12 (9.2%) participants had more than 10 years of experience as certified occupational therapists. Most participants were from Riyadh, Jeddah, and the Eastern Region.

In addition, the questionnaire revealed that the participating occupational therapists were from different clinical settings, such as rehabilitation hospitals, outpatient clinics, and acute care settings.  Table 2 lists the distribution of settings in descending order. In these settings, 54.2% of participating occupational therapists reported working with individuals aged 6–12 years. Only 42% of the participants had worked with patients aged 13–18 years (Table 2).

Furthermore, the results revealed a pattern of frequently observed medical conditions and therapeutic interventions. Participating occupational therapists reported that neurological conditions (64.3% of the responses) were the most frequently observed. Conversely, respiratory diseases were the least frequently reported condition, comprising approximately 4.7% of responses (Table 3). It should be noted that respondents did not identify mental health conditions as an area of their clinical practice.

Furthermore, the survey identified therapeutic interventions employed to address common conditions. As shown in Table 4, therapeutic exercise was the most frequently reported interventional method used by participating occupational therapists, with approximately 77% of responses. Functional electrical stimulation, with only 19.8% of the responses, was the least common interventional method reported.

Regarding the mapping of interventions to the PEO model, the results were as follows: (1) for therapeutic exercises in association with the PEO model, the intervention targeted the person to improve muscle strength, functional performance, and physical ability; (2) the educational interventions focused on the three aspects of the PEO model, that is, the person to optimize problem-solving abilities, occupation at school or workplace, and the environment to modify the surrounding environment. (3) Splinting as an intervention was related to maintaining the joint range of motion, preventing contractures, correcting deformity, and different occupations such as work and school.

Considering the OT framework and processes [36], participating occupational therapists suggested that different types of rehabilitation interventions, such as occupations and activities, education and training, and advocacy, should be applied in OT. When mapping the interventions selected by the sample (Table 5), four aspects of the intervention were covered; however, two aspects were not applied by the therapists including group and virtual intervention. Moreover, respondents covered all five approaches recommended by the OT framework and processes.

## 4. Discussion

In the current study, we aimed to identify the most common disorders managed by occupational therapists currently practicing in Saudi Arabia, as well as the methods used to treat these disorders. This study explored the current practices employed by occupational therapists to address common disorders in rehabilitation clinics in Saudi Arabia. Based on data collected from 131 occupational therapists in Saudi Arabia, neurological conditions were the most common condition encountered in clinical settings, followed by developmental disorders. Therapeutic exercise was the most commonly used intervention, followed by educational intervention.

Herein, our findings revealed several relationships between the reported conditions and therapeutic interventions. According to participating therapists, neurological disorders, including stroke, cerebral palsy, and TBI, were the most frequently observed conditions (~64.3% of respondents). Among interventional strategies reported in the survey, therapeutic exercise, CIMT, proprioceptive neuromuscular facilitation, neurodevelopment treatment, and mirror therapy were the most commonly reported interventional methods for patients with stroke. However, CIMT and neurodevelopment treatment are frequently employed in patients with cerebral palsy. Analyzing the survey results, CIMT and splints were employed in patients with TBI.

Furthermore, 49.6% of occupational therapists ranked developmental disorders second among the most frequently encountered conditions. Specifically, the most common conditions documented by participating therapists were autism, attention deficit hyperactivity disorder, and cerebral palsy, all considered neurological and developmental disorders. Additionally, our analysis revealed that sensory integration interventions were the most commonly used therapeutic interventions for developmental disorders. Children with developmental disorders might be hyper- or hyporesponsive to sensory stimuli. Therefore, challenging children in a joyful, entertaining context would be an ideal approach to ensure that they learn to respond correctly and function normally in their environment. According to the participating therapists, interventions such as educational and counseling sessions for parents and caregivers were also implemented, in addition to environmental and lifestyle changes.

In OT clinics, physical disability was the third most common condition encountered, with spinal cord injury accounting for 44.7% of all cases. Notably, therapeutic exercises were the most frequently used interventional strategies to regain muscle strength and endurance, whereas the use of splints was secondary to preventing deformities. Another treatment approach in patients with spinal cord injury is the use of assistive devices, typically warranting lifetime usage to ease accessibility and participation. The survey also recorded educational interventions, such as counseling and informative sessions for the patient and his family, as an interventional strategy.

In addition to physical disabilities, orthopedic disorders account for 31% of cases observed in OT clinics, with hand injuries being the most frequently documented condition. As examples of orthopedic disorders, participants reported fractures, carpal tunnel syndrome, De Quervain's syndrome, trigger finger, and hip replacement. The most common interventional methods for these conditions include the use of splints, assistive devices, environmental and lifestyle modifications, therapeutic modalities, and exercise.

The relationship between educational qualifications and frequently observed conditions was also examined. A considerable percentage of respondents (88.5%) with bachelor's degrees reported that neurological and developmental conditions were the most frequently encountered in clinical settings. Conversely, therapists with a master's degree, comprising 9.2% of the survey population, predominantly encountered neurological conditions. Orthopedic disorders were the second most common condition reported by participants holding a master's degree.

In addition, data analysis revealed a relationship between the most reported interventions and reported workplaces. Therapeutic exercises were the most commonly reported interventional methods used by therapists in rehabilitation settings, followed by splinting, educational interventions, and therapeutic modalities. Therapeutic exercises are most commonly applied in outpatient clinics, followed by educational interventions and sensory integration therapy. Accordingly, based on survey data analysis, therapeutic exercises were the most therapeutic strategy applied by occupational therapists in Saudi Arabia across different settings. In OT, therapeutic exercise, as the main means of intervention, is used as a preparatory method for occupational performance. Therefore, it is essential to redirect OT services to activities of daily living and use occupation as the main treatment modality. Several challenges, including time constraints, case overload, and therapeutic equipment, impede the proper implementation of novel OT services in Saudi Arabia. Moreover, the biomechanical working paradigm in several health institutions in Saudi Arabia hinders the application of biopsychsocial approaches, such as the therapeutic use of the self or cognitive behavioral therapy [37, 38].

The results of the present study are consistent with those reported previously, revealing that the most common conditions encountered by occupational therapists include stroke, cerebral palsy, and autism [3, 9, 17–20, 25]. TBI was also reported as one of the most common conditions treated by occupational therapists. It should be noted that no previous report has documented TBI as a common condition encountered by occupational therapists.

Compared with the findings of previous reports, our results highlight the application of similar interventional strategies in patients with stroke and autism. Unlike previous results that included the use of bolster swing, ball, ramps, and trampoline in patients with cerebral palsy [18], as reported by Rassafiani et al., our results revealed that neurodevelopmental treatment was the most common intervention for patients with cerebral palsy. Moreover, hippotherapy is a prescribed intervention for individuals with cerebral palsy, as reported by Zadnikar and Kastrin [20]; however, this approach was not reported in our sample.

Gandhi et al. have demonstrated that mirror therapy is a useful and feasible strategy for patients with stroke during the acute, subacute, and chronic disease phases. Additionally, mirror therapy can influence not only motor deficits but also other aspects, such as sensation, visuospatial neglect, and pain [39]. Hence, the results of the current study provide preliminary data supporting the use of mirror therapy in patients with stroke, given that this approach is the most commonly utilized interventional method, as reported by participating occupational therapists.

A large proportion of the participating occupational therapists who had worked with patients with autism reported the efficacy of the sensory integration approach. This finding is consistent with that reported by Schaaf et al. [40]. Reportedly, changes in behavior and participation can be associated with alterations in the ability to process and integrate sensory information for better praxis, providing preliminary evidence supporting the sensory integration approach.

A similar study was conducted in Kuwait by Alenezi and Alsairafi in 2022, though focusing only on the profiling of the pediatric OT practice in Kuwait [41]. Their study indicated that the most commonly seen conditions were developmental delay, learning disability, and neurological disorders. And the most frequently applied intervention techniques were family education, assistive and adaptive equipment prescription, activities of daily living training, and environmental modification [41]. Their finding is in line with the findings of our study, highlighting the similar OT service development and expansion in the region.

As recommended by the AOTA, OT activities should address one of the following domains: occupation and activities, intervention to support occupations, education and training, and advocacy, as highlighted in our research. However, group and virtual interventions were not mentioned in our research sample, although these interventions have been deemed effective in reducing psychological symptoms such as anxiety, increasing positive mood states, and inducing relaxation [3]. Moreover, barriers to establishing and integrating these domains need to be considered, owing to a lack of awareness among occupational therapists regarding their importance and effectiveness within different patient groups. The results of this study illustrate that the approaches used by participating occupational therapists align with the AOTA-recommended interventional approaches, namely, health promotion, restoration (remediation and restoration), maintenance, modification (compensation and adaptation), and prevention. These approaches reflect the comprehensive role of OT rehabilitation implemented by occupational therapists in Saudi Arabia.

The scope of OT practice across Arab countries varies significantly due to differences in healthcare infrastructure, professional regulation, educational standards, and public awareness. In many Arab nations, OT is still an emerging profession, with limited integration into healthcare systems and a narrow focus primarily on physical rehabilitation within hospital settings. Countries such as Egypt and Lebanon have established OT programs yet often face challenges related to professional recognition and standardized licensure, as the program is not established at the academic level [42, 43]. In contrast, Saudi Arabia along with other countries such as Kuwait, Jordan, and Oman had made notable strides in advancing OT practice, supported academic development [29, 44–46]. The country hosts multiple accredited OT educational programs and mandates licensure through the SCFHS, ensuring a regulated professional framework. Therefore, OT is increasingly involved in diverse clinical areas, including pediatrics, neurology, and rehabilitation, although continued efforts are needed to expand services into community and mental health domains [47, 48].

When it comes to the development of new OT services in Saudi Arabia, a culturally sensitive approach is essential. This is specifically true for services relating to the management of mental health and psychological conditions. Cultural knowledge and competency were found to be very important for Middle East OT service development [48]. This is particularly important due to the increased prevalence of mental health disorders in Saudi Arabia [49]. Nearly one in four adults in Saudi Arabia has a lifetime risk of acquiring a diagnosable mental disease, with high rates of social anxiety, obsessive–compulsive disorder, and separation anxiety disorder, particularly in adults [49]. Unfortunately, less than one-third of Saudi Arabians with lifelong psychiatric conditions receive any kind of therapy, indicating a significant treatment gap in the country [49]. Hence, OT services for mental health disorders are also lacking. In an effort to overcome this limitation, more educational campaigns and services should tackle the management of mental health disorders [47]. Awareness about mental health–related OT practice is essential to enhance the profession's presence within this overlooked field.

Moreover, more efforts at the educational level should be implemented to address the gaps in the holistic implementation of OT intervention in Saudi Arabia. Al-Heizan suggested implementing organizational learning within Saudi universities in general and within OT programs in particular [50]. Organizational learning as described by Senge focuses on establishing “organizations where people continually expand their capacity to create the results they truly desire, where new and expansive patterns of thinking are nurtured, where collective aspiration is set free, and where people are continually learning to see the whole together” [51]. Within the OT education, this could be translated into providing educational environments that allow innovations of therapeutic applications and promoting reflection and inquiry where gathering, sharing, embracing, and applying knowledge is enhanced in a dynamic educational environment [50].

### 4.1. Limitations of the Study

One limitation of the current study is the small sample size when compared to the number of licensed occupational therapists in Saudi Arabia. According to the SCFHS, there are about 870 occupational therapists in Saudi Arabia. However, the number of registered occupational therapists may fail to accurately represent the number of working occupational therapists in Saudi Arabia. In addition, a large number of the registered therapists are fresh graduates with minimal experience and do not meet the inclusion criteria of the study. Thus, the results of the current study can be generalized to the sample of participating occupational therapists. Another limitation is the lack of literature resources on this topic, with no published articles assessing the role of OT as the center of rehabilitation interventions in Saudi Arabia. Limited awareness of OT among healthcare professionals in Saudi Arabia remains a significant limitation to the profession's full integration and application. While occupational therapists are increasingly acknowledged as vital members of interdisciplinary rehabilitation teams, OT services largely remain dependent on physician referrals. Thus, greater integration in treatment planning and shared decision-making between physicians and OTs is needed to fully realize the benefits of collaborative care.

### 4.2. Recommendations

Further research is needed to explore OT practices in Saudi Arabia using a larger sample size and more extensive data collection with onsite validation. The results of the current study substantially contribute to the literature on OT practices in Saudi Arabia. These results will be of interest to OT practitioners to identify current treatment approaches administered under different conditions. To the best of our knowledge, this study is the first to address OT practices in Saudi Arabia, and the findings could be a useful reference to raise awareness regarding OT among the public and other healthcare professionals interested in OT practices in Saudi Arabia. This study emphasizes the importance of redirecting the focus of occupational therapists to the core concepts of activities of daily living and the application of occupation as a means and outcome of rehabilitation, which should be highlighted in the clinical and academic settings of OT in Saudi Arabia.

## 5. Conclusions

In the current study, we aimed to identify the most common diseases and treatment methods used by occupational therapists in Saudi Arabia. Based on our results, neurological diseases such as stroke, cerebral palsy, and TBI are the most commonly treated by occupational therapists. Developmental disorders in children, such as autism and cerebral palsy, fall under both neurological and developmental umbrellas. The most common therapeutic interventions for neurological diseases include mirror therapy and CIMT for stroke. CIMT is also used to address TBI and neurodevelopmental treatment of cerebral palsy. Regarding developmental disorders, sensory integration of children and educational services for parents were reported as the most common therapeutic procedures employed by occupational therapists in Saudi Arabia for autism and cerebral palsy. Thus, OTs play a vital role within rehabilitation teams, particularly in managing complex cases of neurological disorders. They are also involved in specialized centers including autism centers, rehabilitation facilities, and day care centers for individuals with disabilities.

## Figures and Tables

**Table 1:** Sociodemographic and educational characteristics of participants.

	**Sections**	**Participants**	**Percentage**
Gender of participants	Female	80	61.1%
	Male	51	38.9%

Education qualifications	Bachelor's degree	117	88.6%
	Master's degree	12	9.1%
	PhD 2	2	1.5%
	Clinical doctorate	1	0.8%

Years of experience	1–3 years	86	64.9%
	4–5 years	25	19.1%
	6–7 years	7	5.3%
	8–10 years	2	1.5%
	Over 10 years	12	9.2%

Regions	Riyadh	85	64.1%
	Jeddah	27	20.6%
	Eastern Province	7	5.3%
	Southern Province	3	2.3%
	Mecca	3	2.3%
	Northern Province	2	1.5%
	Taif	2	1.5%
	Bisha	1	0.8%
	Median	1	0.8%
	Qassim	1	0.8%

**Table 2:** Description of common cases managed by participating occupational therapists.

	**Sections**	**No. of participants**	**Percentage**
Age group of cases	Infants and children < 6	62	47%
	6–12 years	71	53.8%
	13–18 years	55	41.7%
	19–30 years	70	53%
	30–50 years	71	53.8%
	> 50 years	63	47.7%

Setting	Rehabilitation hospital setting	53	40.2%
	Outpatient clinic center	27	20.5%
	Acute care setting	18	13.7%
	Specialized center	12	9.1%
	School setting	9	6.8%
	University clinic	3	2.3%
	Long-term care hospital setting	1	0.8%
	Mental health and addiction	1	0.8%
	Autism center	1	0.8%
	Day care	1	0.8%
	Fitness	1	0.8%
	Outpatient clinics in the hospital	1	0.8%
	Private hospital	1	0.8%
	Early intervention pediatric program	1	0.8%
	Pediatric home healthcare	1	0.8%

**Table 3:** Type of conditions seen by participating occupational therapists.

	**Most frequently seen medical condition**	**N** ** (%)**
1.	Neurological conditions	84 (64.1%)
2.	Developmental disorders	65 (49.6%)
3.	Physical disability	59 (45.0%)
4.	Orthopedic disorders	41 (31.3%)
5.	Sensory-based disabilities	39 (29.8%)
6.	Cognitive disabilities	35 (26.7%)
7.	Diabetes and hypertension	16 (12.2%)
8.	Respiratory diseases	6 (4.5%)

**Table 4:** Interventions applied by participating occupational therapists.

	**Most frequently applied intervention methods**	**N** ** (%)**
1.	Therapeutic exercise	101 (77.1%)
2.	Educational intervention	79 (60.3%)
3.	Splints	74 (56.5%)
4.	Sensory integration therapy	66 (50.4%)
5.	Environment and lifestyle modification	59 (45.0%)
6.	Neurodevelopment treatment (NDT)	57 (43.5%)
7.	Assistive devices and adaptive equipment	56 (42.7%)
8.	Therapeutic modalities (e.g., using ice and hot packs)	53 (40.5%)
9.	Constraint-induced movement therapy (CIMT)	49 (37.4%)
10.	Proprioceptive neuromuscular facilitation (PNF)	32 (24.4%)
11.	Mirror therapy	32 (24.4%)
12.	Functional electrical stimulation	26 (19.8%)

**Table 5:** Mapping of intervention type and approaches to those recommended by AOTA.

**Mapping of intervention type and approaches to those recommended by AOTA**		
	**Applied**	**Not applied**
Intervention type		
Occupation and activities	✓	
Support occupations	✓	
Education	✓	
Training	✓	
Advocacy	✓	
Group intervention		✗
Virtual intervention		✗
Approaches to intervention		
Health promotion	✓	
Restore (remediation, restoration)	✓	
Maintain	✓	
Modify (compensation, adaptation)	✓	
Prevention	✓	

## Data Availability

The data used to support the findings of the study are available on request from the first author.

## References

[1] The American Occupational Therapy Association (2010). *Occupational Therapy Part of Health Care Professionals*.

[2] Trembath F., Dahl-Popolizio S., Vanwinkle M., Milligan L. (2019). Retrospective Analysis: Most Common Diagnoses Seen in a Primary Care Clinic and Corresponding Occupational Therapy Interventions. *Open Journal of Occupational Therapy*.

[3] Leland N. E., Fogelberg D. J., Halle A. D., Mroz T. M. (2017). Occupational Therapy and Management of Multiple Chronic Conditions in the Context of Health Care Reform. *American Journal of Occupational Therapy*.

[4] American Occupational Therapy Association (2021). Occupational Therapy Scope of Practice. *American Journal of Occupational Therapy*.

[5] World Federation of Occupational Therapists (2012). Definition of Occupational Therapy. https://wfot.org/about-occupational-therapy.

[6] Legg L. A., Lewis S. R., Schofield-Robinson O. J., Drummond A., Langhorne P. (2017). Occupational Therapy for Adults With Problems in Activities of Daily Living After Stroke. *Cochrane Database of Systematic Reviews*.

[7] De Coninck L., Bekkering G. E., Bouckaert L., Declercq A., Graff M. J. L., Aertgeerts B. (2017). Home- and Community-Based Occupational Therapy Improves Functioning in Frail Older People: A Systematic Review. *Journal of the American Geriatrics Society*.

[8] Wenborn J., O’Keeffe A. G., Mountain G. (2021). Community Occupational Therapy for People With Dementia and Family Carers (COTiD-UK) Versus Treatment as Usual (Valuing Active Life in Dementia [VALID]) Study: A Single-Blind, Randomised Controlled Trial. *PLoS Medicine*.

[9] Ezekiel L., Feaver S., Duncan E. A. S. (2020). Application of Theoretical Approaches to Movement and Cognitive-Perceptual Dysfunction Within Occupation-Focused Practice. *Foundations for Practice in Occupational Therapy*.

[10] Abu Tariah H., Aljehani A. S., Alenazi D. Y., Alturaif D. A., Alsarhani M. N. (2020). Occupational Therapy Goal Achievement for People With Stroke: A Retrospective Study. *Occupational Therapy International*.

[11] Langer D., Luria S., Maeir A., Erez A. (2014). Occupation-Based Assessments and Treatments of Trigger Finger: A Survey of Occupational Therapists From Israel and the United States. *Occupational Therapy International*.

[12] Colbourn J., Heath N., Manary S., Pacifico D. (2008). Effectiveness of Splinting for the Treatment of Trigger Finger. *Journal of Hand Therapy*.

[13] Lunsford D., Valdes K., Hengy S. (2019). Conservative Management of Trigger Finger: A Systematic Review. *Journal of Hand Therapy*.

[14] Choudhury M. M., Tay S. C. (2014). Prospective Study on the Management of Trigger Finger. *Hand Surgery*.

[15] Uyanik M., Bumin G., Kayihan H. Ü. L. Y. A. (2003). Comparison of Different Therapy Approaches in Children With Down Syndrome. *Pediatrics International*.

[16] Ciucurel C., Iconaru E. I. (2012). Occupational Therapy for Children With Down Syndrome–A Case Study. *Procedia-Social and Behavioral Sciences*.

[17] Raj S., Stanley M., Mackintosh S., Fryer C. (2020). Scope of Occupational Therapy Practice for Adults With Both Down Syndrome and Dementia: A Cross-Sectional Survey. *Australian Occupational Therapy Journal*.

[18] Rassafiani M., Akbarfaimi N., Hosseini S. A., Shahshahani S., Karimlou M., Tabatabai Ghomsheh F. (2020). The Effect of the Combination of Active Vestibular Interventions and Occupational Therapy on Balance in Children With Bilateral Spastic Cerebral Palsy: A Pilot Randomized Controlled Trial. *Iranian Journal of Child Neurology*.

[19] Figueiredo P. R., Mancini M. C., Feitosa A. M. (2020). Hand–Arm Bimanual Intensive Therapy and Daily Functioning of Children With Bilateral Cerebral Palsy: A Randomized Controlled Trial. *Developmental Medicine & Child Neurology*.

[20] Zadnikar M., Kastrin A. (2011). Effects of Hippotherapy and Therapeutic Horseback Riding on Postural Control or Balance in Children With Cerebral Palsy: A Meta-Analysis. *Developmental Medicine & Child Neurology*.

[21] Christie L., Inman J., Davys D., Cook P. A. (2021). A Systematic Review Into the Effectiveness of Occupational Therapy for Improving Function and Participation in Activities of Everyday Life in Adults With a Diagnosis of Depression. *Journal of Affective Disorders*.

[22] Jones M. J. (2025). A Cognitive Behavioral Approach to Improving Performance and Satisfaction in Meaningful Occupations in the Outpatient Mental Health Occupational Therapy Setting. *Occupational Therapy in Mental Health*.

[23] Rouse J., Hitch D. (2014). Occupational Therapy Led Activity Based Group Interventions for Young People With Mental Illness: A Literature Review. *New Zealand Journal of Occupational Therapy*.

[24] Castro D., Dahlin-Ivanoff S., Mårtensson L. (2014). Occupational Therapy and Culture: A Literature Review. *Scandinavian Journal of Occupational Therapy*.

[25] Zango Martín I., Flores Martos J. A., Moruno Millares P., Björklund A. (2015). Occupational Therapy Culture Seen Through the Multifocal Lens of Fieldwork in Diverse Rural Areas. *Scandinavian Journal of Occupational Therapy*.

[26] Zango-Martín I., Nafai S., El Ouazzani S., Derkaoui J., Stevens-Nafai E., Codern-Bové N. (2022). Understanding the Role and Importance of Occupational Therapy in Mental Health Services in Morocco: Perspectives From Mental Health Professionals. *Work*.

[27] Ministry of Health (2022). *Statistical Yearbook-Part 4: Health Activities 2022*.

[28] Aljabri N. Q., Bulkeley K., Cusick A. (2024). The Occupational Therapy Profession in Saudi Arabia. *Occupational Therapy International*.

[29] Al-Heizan M. O., Alhammad S. A., Aldaihan M. M., Alwadeai K. S. (2023). Occupational Therapy Education in Saudi Arabia: Barriers and Solutions From a Cross-Sectional Survey Study. *Cureus*.

[30] Meny A. H., Hayat A. A., Ain Q. U. (2021). Knowledge About Occupational Therapy Among People in Saudi Arabia. *Journal of Evolution of Medical and Dental Sciences*.

[31] Meny A. H., Hayat A. A. (2017). Knowledge About Occupational Therapy in Makkah, Saudi Arabia. Where Do Health Care Professionals Stand. *International Annals of Medicine*.

[32] Sarsak H. I. (2020). Perceptions of the Occupational Therapy Profession Among Medical and Health Science Students in Saudi Arabia. *Annals of International Occupational Therapy*.

[33] Banamah A., Meny A., Eldigire M., Qurban W. (2025). Knowledge of the Differences Between Occupational Therapy and Physical Therapy Among the General Population in Saudi Arabia. *Journal of Public Health*.

[34] Vadivel S., Mani P., Al Anezi N. (2021). Knowledge and Awareness About Occupational Therapy Among Healthcare Professionals in Al-Ahsa. *World Journal of Biology Pharmacy and Health Sciences*.

[35] Strong S., Rigby P., Stewart D., Law M., Letts L., Cooper B. (1999). Application of the Person-Environment-Occupation Model: A Practical Tool. *Canadian Journal of Occupational Therapy*.

[36] Boop C., Cahill S. M., Davis C. (2020). Occupational Therapy Practice Framework: Domain and Process Fourth Edition. *AJOT: American Journal of Occupational Therapy*.

[37] Almalki M., FitzGerald G., Clark M. (2011). *Health Care System in Saudi Arabia: An Overview*. *Eastern Mediterranean Health Journal*.

[38] AlHadi A. N., Alammari K. A., Alsiwat L. J. (2021). Perception of Mental Health Care Professionals in Saudi Arabia on Computerized Cognitive Behavioral Therapy: Observational Cross-Sectional Study. *JMIR Formative Research*.

[39] Gandhi D. B., Sterba A., Khatter H., Pandian J. D. (2020). Mirror Therapy in Stroke Rehabilitation: Current Perspectives. *Therapeutics and Clinical Risk Management*.

[40] Schaaf R. C., Hunt J., Benevides T. (2012). Occupational Therapy Using Sensory Integration to Improve Participation of a Child With Autism: A Case Report. *American Journal of Occupational Therapy*.

[41] Alenezi A., Alsairafi S. (2022). Profile of Pediatric Occupational Therapy Practice in Kuwait: A Qualitative Study. *Scholars Journal of Applied Medical Sciences*.

[42] Amireh M. (2019). Occupational Therapy Practice in Egypt Cultural Perspective. *Global Journal of Intellectual & Developmental Disabilities*.

[43] Saleh N. E. H., Summaka M., Zein H. (2024). ICF-Based Multidisciplinary Approach to Rehabilitation of People With Disabilities: Perspective and Current Practices Within the Health, Rehabilitation, Integration, and Research Center in Lebanon. *Discover Health Systems*.

[44] Alotaibi N. M., Manee F. S., Murphy L. J., Rassafiani M. (2019). Knowledge About and Attitudes of Interdisciplinary Team Members Toward Occupational Therapy Practice: Implications and Future Directions. *Medical Principles and Practice*.

[45] Darawsheh W. B. (2018). Awareness and Knowledge About Occupational Therapy in Jordan. *Occupational Therapy International*.

[46] Al Busaidy N. S. M., Borthwick A. (2012). Occupational Therapy in Oman: The Impact of Cultural Dissonance. *Occupational Therapy International*.

[47] Alodan H. A., Squire R. E. (2022). Emerging Occupational Therapy in Mental Health Practice in Saudi Arabia: A Qualitative Study. *Occupational Therapy in Mental Health*.

[48] Awaad T. (2003). Culture, Cultural Competency and Psychosocial Occupational Therapy: A Middle Eastern Perspective. *British Journal of Occupational Therapy*.

[49] Chatterji S. (2020). The Saudi National Mental Health Survey: Filling Critical Gaps in Methodology and Data in Mental Health Epidemiology. *International Journal of Methods in Psychiatric Research*.

[50] Al-Heizan M. O. (2023). Learning Organizations in Saudi Universities: Implications for Occupational Therapy Education. *Journal of Taibah University Medical Sciences*.

[51] Senge P. M. (2006). *The Fifth Discipline: The Art and Practice of The Learning Organization*.

